# Inertia location and slow network modes determine disturbance propagation in large-scale power grids

**DOI:** 10.1371/journal.pone.0213550

**Published:** 2019-03-21

**Authors:** Laurent Pagnier, Philippe Jacquod

**Affiliations:** 1 School of Engineering, University of Applied Sciences of Western Switzerland HES–SO, Sion, Switzerland; 2 Institute of Theoretical Physics, EPFL, Lausanne, Switzerland; 3 Department of Quantum Matter Physics, University of Geneva, Geneva, Switzerland; Wuhan University, CHINA

## Abstract

Conventional generators in power grids are steadily substituted with new renewable sources of electric power. The latter are connected to the grid via inverters and as such have little, if any rotational inertia. The resulting reduction of total inertia raises important issues of power grid stability, especially over short-time scales. With the motivation in mind to investigate how inertia reduction influences the transient dynamics following a fault in a large-scale electric power grid, we have constructed a model of the high voltage synchronous grid of continental Europe. To assess grid stability and resilience against disturbance, we numerically investigate frequency deviations as well as rates of change of frequency (RoCoF) following abrupt power losses. The magnitude of RoCoF’s and frequency deviations strongly depend on the fault location, and we find the largest effects for faults located on the support of the slowest mode—the Fiedler mode—of the network Laplacian matrix. This mode essentially vanishes over Belgium, Eastern France, Western Germany, northern Italy and Switzerland. Buses inside these regions are only weakly affected by faults occuring outside. Conversely, faults inside these regions have only a local effect and disturb only weakly outside buses. Following this observation, we reduce rotational inertia through three different procedures by either (i) reducing inertia on the Fiedler mode, (ii) reducing inertia homogeneously and (iii) reducing inertia outside the Fiedler mode. We find that procedure (iii) has little effect on disturbance propagation, while procedure (i) leads to the strongest increase of RoCoF and frequency deviations. This shows that, beyond absorbing frequency disturbances following nearby faults, inertia also mitigates frequency disturbances from distant power losses, provided both the fault and the inertia are located on the support of the slowest modes of the grid Laplacian. These results for our model of the European transmission grid are corroborated by numerical investigations on the ERCOT transmission grid.

## 1 Introduction

The short-time voltage angle and frequency dynamics of AC power grids is standardly modeled by the swing equations [1]. The latter determine how local disturbances about the synchronous operational state propagate through the grid. They emphasize in particular how voltage angle and frequency excursions are partially absorbed on very short time scales by the inertia of rotating machines, before primary control sets in. With the energy transition, more and more new renewable energy sources (RES) such as solar photovoltaic units—having no inertia—and wind turbines—whose inertia is at this time essentially suppressed by inverters—substitute for conventional power generators. The resulting overall reduction in rotational inertia raises a number of issues related to system dynamics and stability [2, 3]. It is in particular desirable to determine how much inertia is sufficient and where to optimally locate it to guarantee short-time grid stability. Determining the optimal placement of inertia is of paramount importance at the current stage of the energy transition, as it would help determine where the substitution of conventional generators by RES crucially needs to be accompanied by the deployment of synchronous condensers or synthetic inertia.

The impact of lowered levels of inertia on grid stability has been investigated in a number of papers. Gautam et al. [4] and Eftekharnejad et al. [5] emphasized an interesting correlation between the location of inertia reduction and specific electromechanical modes in the case of increased wind turbine and photovoltaic penetration respectively. Ulbig et al. investigated the impact of reduced inertia on power system stability for a two-area model [2]. Extended to three-area systems their analysis led them to postulate that, at fixed amount of inertia, meshed grids have a greater resilience to disturbances than unmeshed ones [6]. These works further raised the issue of optimal inertia placement in a grid with reduced total amount of inertia. This issue is interesting from the point of view of synthetic inertia, obtained by controlling the inverters connecting RES to the grid [7–9] and which can in principle be deployed where needed. It is moreover crucial to anticipate where the substitution of conventional power generators would require significant inertia compensation and where not. Borsche et al. evaluated damping ratios and transient overshoots to optimize the placement of virtual inertia [10]. Poolla et al. proposed a different placement optimization based on the minimization of $H_{2}$ norms [11], while Pirani et al. adopted an approach based on $H_{\infty }$ norms [12]. As pointed out by Borsche and Dörfler [13], the objective functions to be minimized in these works are not directly related to the standard operational criteria of Rate of Change of Frequency (RoCoF) or frequency deviations in electric power grids. To bridge that gap, Ref. [13] constructed an inertia placement optimization algorithm based on these criteria.

In this paper we investigate RoCoF’s under abrupt power losses in high voltage power grids. We set the fault magnitude at Δ*P* = 900 MW, large enough to generate a significant response but small enough that it is smaller or equal to the generation capacity of many power plants distributed all over continental Europe. This allows us to compare similar faults at various locations and to directly relate discrepancies in the ensuing disturbance propagation to the geographical location of the fault. By the end of the manuscript we briefly consider larger faults of Δ*P* = 3000 MW corresponding to the ENTSO-E reference incident [14]. Our goal is to understand how the ensuing disturbance propagates through the system, as a function of the power fault location. To that end we construct a model of the synchronous high voltage grid of continental Europe that includes geolocalization, dynamical parameters and rated voltage of all buses, as well as electrical parameters of all power lines. Our approach is mostly numerical and therefore is not limited by assumptions of constant inertia or damping coefficients that are necessary to obtain analytical results. Our model is unique in that it is based on a realistic map of the distribution of rotational inertia in the synchronous European grid. Disturbance propagation under noisy perturbations have been investigated in a number of works on dynamical networks (see e.g. Ref. [15–17]). What makes the present work special is the spatio-temporal resolution of our investigations on large-scale networks, which allows us to correlate the impact of the location of the fault with the nonhomogeneous distribution of inertia and the spatial support of the slowest modes of the network Laplacian.

**Fig 1:**
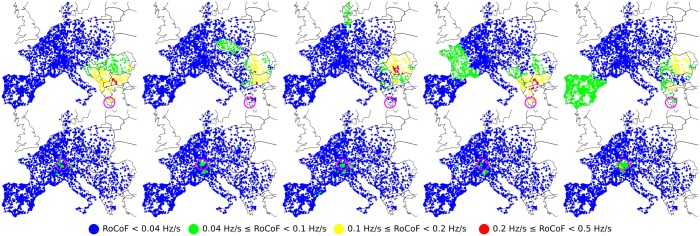
Spatio-temporal evolution of local RoCoFs for two different power losses of Δ*P* = 900 MW in a moderate load (typical of a standard summer evening) configuration of the synchronous grid of continental Europe of 2018. The top five panels correspond to a fault in Greece and the bottom five to a fault in Switzerland. In both cases, the fault location is indicated by a purple circle. Panels correspond to snapshots over time intervals 0-0.5[s], 0.5-1[s], 1-1.5[s], 1.5-2[s] and 2-2.5[s] from left to right.

We numerically simulate sudden power losses at different locations on the grid for various loads. Using the swing equations, we evaluate how the resulting frequency disturbance propagates through the grid by recording RoCoF’s at all buses. This is illustrated in Fig 1 for two different fault locations of the same magnitude, Δ*P* = 900 MW. This relatively moderate fault (on the scale of the European grid) generates a significant response, with RoCoF’s reaching 0.5 Hz/s, over large areas for a power loss in Greece. On the other hand, RoCoF’s never exceed 0.1 Hz/s when a fault of the same magnitude occurs in Switzerland. After a systematic investigation of faults over the whole grid, we relate these differences in behavior to (i) the local inertia density in the area near the fault at times *t* ≲ 1 − 2 s and (ii) the amplitude of the slowest modes of the grid Laplacian on the faulted bus for times *t* ≳ 1 − 2 s. Point (i) is expected and well known, however point (ii) is, to the best of our knowledge, a new observation. It has in particular the surprising consequence that, when the slowest modes have disconnected structures as is the case of the European grid, frequency disturbances propagate between distant areas, almost without affecting some areas in between. Comparing different scenarios for inertia withdrawal, corresponding to substituting new RES for conventional power plants in different regions, we find that inertia withdrawal from areas with large components of the slowest modes of the grid Laplacian results in significantly higher RoCoF’s. This has important consequences for planning and optimal inertia location in future low-inertia power grids.

This manuscript is organized as follows. In Section 2 we give some details of our model and approach. Section 3 presents numerical investigations of RoCoF’s under abrupt power losses at different locations on the power grid. It relates the magnitude of the response to such faults to the location of the fault, in particular the amplitude on the slowest Laplacian modes on the faulted bus. Section 4 amplifies on Section 3 by investigating the effect of reducing the inertia on different areas of the grid. We summarize our findings and results in the Conclusion Section. Details on the model and further numerical results are presented in the Appendix.

## 2 Transmission grid model

We have imported and combined publicly available data to construct a geolocalized model of the high voltage synchronous grid of continental Europe. The geographical location and the electrical parameters of each bus is determined, including voltage level, dynamical parameters (inertia and damping coefficients), generator type and rated power. Line capacities are extracted from their length. They are compared with known values for a number of lines and found to be in good agreement. Different load situations are investigated using a demographically-based distribution of national loads, together with a dispatch based on a DC optimal power flow. Details of these procedures are given in S2 Appendix. To confirm our conclusions, we alternatively used a model of the Texas ERCOT transmission grid [18], where inertia and damping coefficients are obtained using the same procedure as for the European model.

The models are treated within the lossless line approximation [1], where the electrical power $P_{i}^{e}$ injected or extracted at bus #*i* is related to the voltage phase angles {*θ*_*i*_} as

$$\begin{array}{c} P_{i}^{e}=\sum_{j\in V}B_{ij}\;V_{i}V_{j}\;\text{sin}(\theta_{i}-\theta_{j})\;. \end{array}$$ (1)

Here, *B*_*ij*_ gives the imaginary part of the admittance of the power line connecting bus #*i* at voltage *V*_*i*_ to bus #*j* at voltage *V*_*j*_ and V is the set of the *N* buses in the system. Voltages are assumed constant, $V_{i}=V_{i}^{\left(0\right)}$ and are equal to either 220 or 380 kV. We denote $V_{\text{gen}}\subset V$ the subset of buses corresponding to generator buses. Their dynamics is described by the swing equations [1, 19]

$$\begin{array}{c} m_{i}{\dot{\omega }}_{i}+d_{i}\omega_{i}=P_{i}^{\left(0\right)}-P_{i}^{e}\;,\;\text{if}\;i\in V_{\text{gen}}\;, \end{array}$$ (2)

where $\omega_{i}={\dot{\theta }}_{i}$ is the local voltage frequency, *m*_*i*_ and *d*_*i*_ are the inertia and damping coefficients of the generator at bus *i* respectively. The complement subset $V_{\text{load}}=V\backslash V_{\text{gen}}$ contains inertialess generator or consumer buses with frequency dependent loads [19] and a dynamics determined by the swing equations [1, 19]

$$\begin{array}{c} d_{i}\omega_{i}=P_{i}^{\left(0\right)}-P_{i}^{e}\;,\;\text{if}\;i\in V_{\text{load}}\;. \end{array}$$ (3)

In (2) and (3), $P_{i}^{\left(0\right)}$ gives the power input ($P_{i}^{\left(0\right)}>0$) at generator buses or the power output ($P_{i}^{\left(0\right)}<0$) at consumer buses prior to the fault. We consider that (2) and (3) are written in a rotating frame with the rated frequency of *ω*_0_ = 2*πf* with *f* = 50 or 60 Hz, in which case $\sum_{i}P_{i}^{\left(0\right)}=0$.

In order to investigate transient dynamics following a plant outage, we consider abrupt power losses $P_{i}^{\left(0\right)}\to P_{i}^{\left(0\right)}-\Delta P$ with Δ*P* = 900 MW on the European grid model and 500 MW on the ERCOT grid model. In both cases, a single plant is faulted and only power plants with *P*_*i*_ ≥ Δ*P* can be faulted. The values of Δ*P* are chosen so that many contingencies with different locations homogeneously distributed over the whole grid can be investigated. Larger faults with significantly larger RoCoF’s will be briefly discussed in the Conclusion Section. Frequency changes are then calculated from (2) and (3), with initial conditions given by their stationary solution and the faulted bus #*b* treated as a load bus with power injection $P_{b}=P_{b}^{\left(0\right)}-\Delta P$, vanishing inertia, *m*_*b*_ = 0, and unchanged damping coefficient *d*_*b*_. This should be considered as our definition of a fault, where for each faulted generator, the same amount of power is always lost, together with the full inertia of the faulted generator.

## 3 Disturbance propagation

Our numerical data monitor the voltage angle and frequency excursion following an abrupt power loss. Fig 1 shows two such events with series of snapshots illustrating the propagation of the disturbance over the continental European grid during the first 2.5 seconds after the contingency. The two events differ only by the location of the power loss. In the top row the faulted power plant is in Greece, while in the bottom row it is in Switzerland (fault locations are indicated by purple circles). In both instances, the lost power is Δ*P* = 900 MW and the grid, including loads and feed-ins, inertia distribution, damping parameters and electrical parameters of all power lines, is the same.

**Fig 2:**
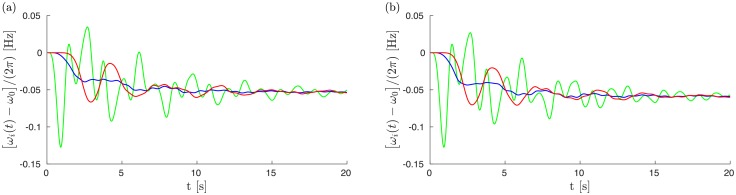
Frequency deviations as a function of time for the fault illustrated (a) in the top row of Fig 1 and (b) in the top row of Fig 5 [with inertia in France reduced by a factor of two compared to panel (a)], for three buses in the Balkans (green), France (blue) and Spain (red).

The two disturbance propagations shown are dramatically different. For a fault in Greece, RoCoF’s reach 0.5 Hz/s for times up to 2s. The disturbance furthermore propagates across almost all of the grid. Quite surprisingly, it seems to jump from Germany to Spain while avoiding Eastern France, Belgium and Switzerland in between. We have checked that this is not an artifact of the way we plot the average RoCoF, but truly reflects a moderate effect on the local grid frequency in those regions. This is illustrated in Fig 2(a) which shows frequency deviations for three buses in the Balkans, in Eastern France and in Spain for the fault in the top row of Fig 1. While the Balkanic and Spanish buses oscillate rather strongly, the french bus displays weak oscillations about a frequency reduction reflecting the loss of power generation Δ*P*. Also remarkable is the RoCoF persistence in eastern Europe at later times, *t* > 2 s.

**Fig 3:**
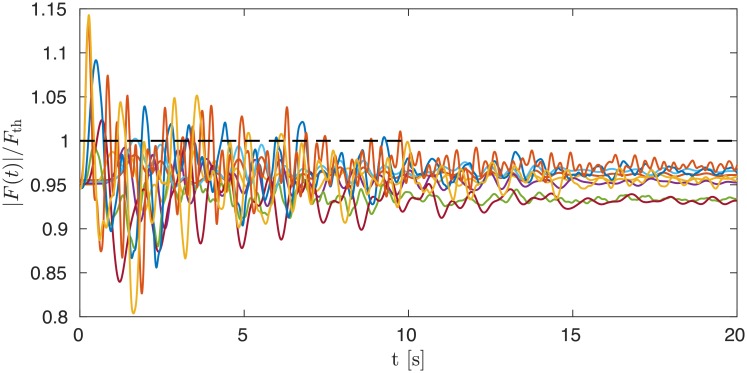
Load flows *F*(*t*) on ten of the originally most heavily loaded lines that exhibit the largest response to the same fault as in the top row of Fig 1. In all cases, flows are normalized by their thermal limit *F*_th_ which varies from line to line.

Fig 3 shows load flow oscillations on the ten power lines in the network that exhibit the largest response to the same fault as in the top row of Fig 1. The flows exceed their thermal limits by almost 15% in several cases, however they quickly fall back below their thermal limit, which they never exceed for times longer than 10 seconds. Accordingly one does not expect any cascade of failures triggered by line faults in the cases considered here of power losses of 900 MW.

For a fault in Switzerland, on the other hand, RoCoF’s never exceed 0.1 Hz/s and the disturbance does not propagate beyond few hundred kilometers. We have systematically investigated disturbance propagation for faults located everywhere on the European grid model and found that major discrepancies between fault located in the Portugal-Spain area or the Balkans generate significantly stronger and longer disturbances, propagating over much larger distances than faults located in Belgium, Eastern France, Western Germany or Switzerland.

**Fig 4:**
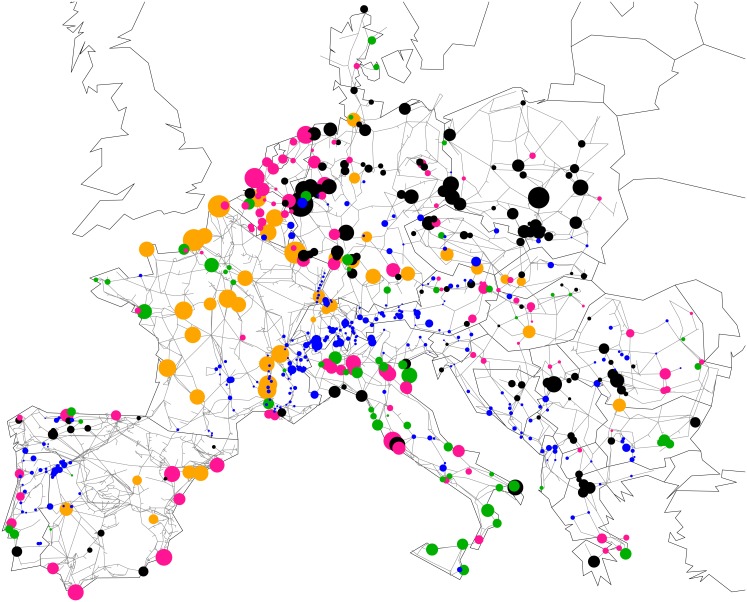
Inertia parameters of generators in our model of the synchronous grid of continental Europe. The disk size is proportional to *m*_*i*_ and the colors label hydro (blue), nuclear (orange), gas (pink), coal (black) and other (green) power plants.

This discrepancy in behaviors is partly due to the distribution of inertia in the European grid. As a matter of fact, the latter is not homogeneous, as is shown in Fig 4. Inertia density is smaller in Spain and Eastern Europe and larger in a strip from Belgium to Northern Italy, including France, Western Germany and Switzerland. Inertia is not only position-dependent, it is also time-dependent as it is directly related to the rotating machines connected to the grid at any given time [2, 6]. Our results below are obtained both for a typical summer evening (with moderate load and thus reduced total inertia) and a typical winter evening (with large load and thus larger total inertia).

**Fig 5:**
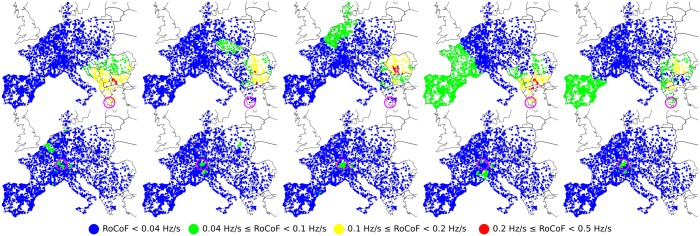
Spatio-temporal evolution of local RoCoFs for two different power losses of Δ*P* = 900 MW in a moderate load (typical of a standard summer evening) configuration of the synchronous grid of continental Europe of 2018. The top five panels correspond to a fault in Greece and the bottom five to a fault in Switzerland. In both cases, the fault location is indicated by a purple circle. Panels correspond to snapshots over time intervals 0-0.5[s], 0.5-1[s], 1-1.5[s], 1.5-2[s] and 2-2.5[s] from left to right. The situation is the same as in Fig 1, except that all inertia coefficients *m*_*i*_ in France have been divided by two.

**Fig 6:**
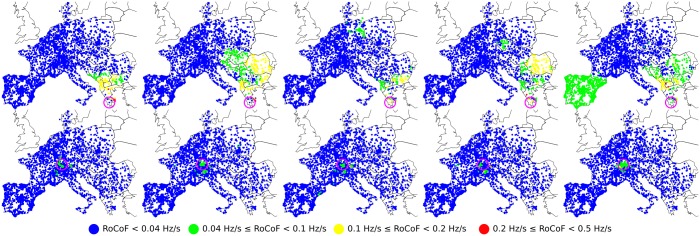
Spatio-temporal evolution of local RoCoFs for two different power losses of Δ*P* = 900 MW in a moderate load (typical of a standard summer evening) configuration of the synchronous grid of continental Europe of 2018. The top five panels correspond to a fault in Greece and the bottom five to a fault in Switzerland. In both cases, the fault location is indicated by a purple circle. Panels correspond to snapshots over time intervals 0-0.5[s], 0.5-1[s], 1-1.5[s], 1.5-2[s] and 2-2.5[s] from left to right. The situation is the same as in Fig 1, except that all inertia coefficients *m*_*i*_ in the Balkans have been multiplied by two.

To investigate the influence of inertia distribution on frequency disturbance propagation, we simulated the same faults as in Fig 1, first, artificially reducing inertia by a factor of two in France, second artificially increasing inertia by a factor of two in the Balkans. The results are shown in Figs 5 and 6 respectively. First, one sees in Fig 5 that reducing the inertia in France has only a local effect. Frequency disturbance from a nearby fault in Switzerland propagates over a larger distance with reduced inertia in France, as expected, however there is very little effect on disturbance propagation from a power loss in Greece. Particularly interesting is that even with a two-fold reduction of inertia in France, there is no increase of the disturbance affecting Eastern France and only a mild increase of it in Western France from a power loss in Greece. Frequency deviations for three buses in the Balkans, in Eastern France and in Spain are shown in Fig 2(b). Despite the reduced inertia in France, the french bus still displays only weak oscillations of frequency about an average frequency decrease characteristic of power losses. The overall frequency evolution is surprisingly almost indistinguishable from the case with normal inertia in France in Fig 2(a). Second, increasing the inertia in the Balkans certainly absorbs part of the frequency disturbance from a nearby fault in Greece. This can be seen in Fig 6. However, relatively large RoCoF’s still persists at *t* > 2 s, with a magnitude that is reduced only because the initial disturbance has been partially absorbed by the increased inertia at short times, *t* < 1 s. We therefore conclude that strong discrepancies in frequency disturbance propagation depending on the location of an abrupt power loss cannot be understood solely on the basis of inertia distribution. In particular, (i) it is not inertia that renders France almost immune to frequency disturbance generated by a power loss in Eastern Europe or Spain, (ii) it is not only the lack of inertia in the Balkans that allows the persistance there of relatively large RoCoF’s at *t* > 2s. Figs A and B in S1 Appendix further show similar behaviors in disturbance propagation following different faults or under different initial load configurations.

We can gain some qualitative understanding into these phenomena through spectral graph theory under simplifying assumptions on model parameters. From (2) and (3) and for small angle differences between connected buses in the initial operational state, weak angle deviations have a dynamics governed by

$$\begin{array}{c} M\dot{\omega }+D\omega =P-L\;\theta \;, \end{array}$$ (4)

with the diagonal matrices ***M*** = diag({*m*_*i*_}) (*m*_*i*_ ≠ 0 for generators only), ***D*** = diag({*d*_*i*_}) and the Laplacian matrix ***L*** of the grid, with elements ${\left(L\right)}_{ij}=-B_{ij}V_{i}^{\left(0\right)}V_{j}^{\left(0\right)}$, *i* ≠ *j* and ${\left(L\right)}_{ii}=\sum_{k}B_{ik}V_{i}^{\left(0\right)}V_{k}^{\left(0\right)}$. Voltage angles and frequencies as well as power injections have been cast into vector form in (4), i.e. ***θ*** = (*θ*_1_, …*θ*_*N*_) and so forth. The Laplacian matrix is real and symmetric, as such it has a complete orthogonal set of eigenvectors {***u***_1_, …, ***u***_*N*_} with eigenvalues {λ_1_, …λ_*N*_}. The zero row and column sum property of ***L*** implies that λ_1_ = 0, corresponding to an eigenvector with constant components, ${\left(u_{1}\right)}^{⊤}=\left(1,\ldots ,1\right)/\sqrt{N}$. If the grid is connected, as in our case, all other eigenvalues of ***L*** are strictly positive, λ_*α*_ > 0 for *α* = 2, …, *N*. This guarantees linear stability of fixed point solutions to (4) in the sense that small angle and frequency deviations are exponentially damped with time.

Our initial state is a stationary state of (4), characterized by *ω*_*i*_ = 0 ∀*i* (since we work in the rotating frame) and a set of voltage angles ***θ***^(0)^. We then let this initial state evolve under (4) after a power loss with *P*_*b*_ → *P*_*b*_ − Δ*P* at bus # *b*. Using the method of Ref. [20, 21] we can compute frequency deviations at bus #*i* and time *t* as a spectral sum over the eigenvectors, {***u***_*α*_}, and eigenvalues, {λ_*α*_} of the Laplacian matrix, under the assumption of homogeneous inertia and damping coefficients, *m*_*i*_ = *m* and *d*_*i*_ = *d*. One gets, with *γ* = *d*/*m*,

$$\delta \omega_{i}\left(t\right)=\frac{\Delta P\;e^{-\gamma t/2}}{m}\sum_{\alpha =1}^{N}u_{\alpha i}u_{\alpha b}\frac{\text{sin}(\sqrt{\lambda_{\alpha }/m-\gamma^{2}/4}\;t)}{\sqrt{\lambda_{\alpha }/m-\gamma^{2}/4}}\;.$$ (5)

In real power grids frequencies are monitored at discrete time intervals *t* → *k*Δ*t*, with Δ*t* ranging between 40 ms and 2 s [22]. RoCof’s are then evaluated as the frequency slope between two such measurements. In our numerics, we use Δ*t* = 0.5 s. The RoCoF at bus #*i* reads

$$\begin{array}{c} r_{i}\left(t\right)=\frac{\delta \omega_{i}(t+\Delta t)-\delta \omega_{i}(t)}{2\pi \Delta t}\;. \end{array}$$ (6)

Together with (5), this gives

$$\begin{array}{ccc} r_{i}\left(t\right) & = & \frac{\Delta P\;e^{-\gamma t/2}}{2\pi m}\sum_{\alpha =1}^{N}\frac{u_{\alpha i}u_{\alpha b}}{\sqrt{\lambda_{\alpha }/m-\gamma^{2}/4}\;\Delta t}\\ & \times & [e^{-\gamma \Delta t/2}\;\text{sin}(\sqrt{\lambda_{\alpha }/m-\gamma^{2}/4}\;\left(t+\Delta t\right))\\ & & -\text{sin}(\sqrt{\lambda_{\alpha }/m-\gamma^{2}/4}\;t)]\;. \end{array}$$ (7)

**Fig 7:**
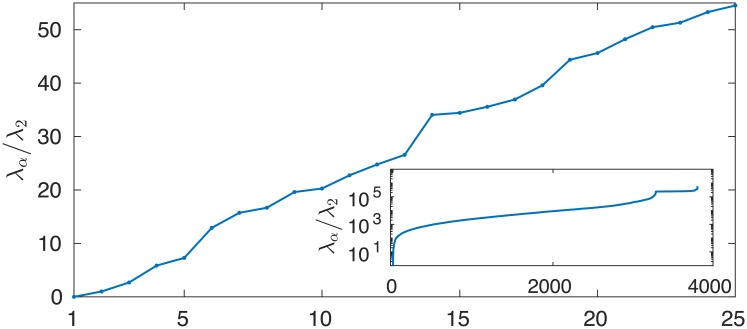
Normalized spectrum λ_*α*_/λ_2_ of the network Laplacian for our model of the high voltage synchronous grid of continental Europe.

The term *α* = 1 gives a position-independent contribution to the RoCoF, $r_{i}^{\left(1\right)}\left(t\right)=\Delta P\;e^{-\gamma t}\left(1-e^{-\gamma \Delta t}\right)/2\pi mN\gamma \Delta t$. It is maximal and inversely proportional to the inertia coefficient *m* at short times, $r_{i}^{\left(1\right)}\left(t\to 0\right)\to \Delta P/2\pi mN$. All other terms *α* > 1 have oscillations with both amplitude and period depending on $\sqrt{\lambda_{\alpha }/m-\gamma^{2}/4}$. High-lying eigenmodes with large *α* and large eigenvalues λ_*α*_ therefore contribute much less than low-lying eigenmodes, both because their oscillation amplitude is reduced and because they oscillate faster, which leads to faster cancellation of terms. With our choice of Δ*t* = 0.5 s we find $\sqrt{\lambda_{\alpha }/m-\gamma^{2}/4}\Delta t\in \left[0.54,416\right]$ for *α* > 1 in our model. The second lowest value is $\sqrt{\lambda_{3}/m-\gamma^{2}/4}\Delta t=0.89$, almost twice larger than the first one. Fig 7 shows the eigenvalues of the network Laplacian, in particular how they quickly increase in value in the lower part of the spectrum and how their density increases as one reaches the middle part of the spectrum. Accordingly, one expects that only few eigenmodes of the network Laplacian, corresponding to its lowest nonvanishing eigenvalues, effectively matter in the spectral sum in (7). Higher-lying modes have only short-lived contributions.

**Fig 8:**
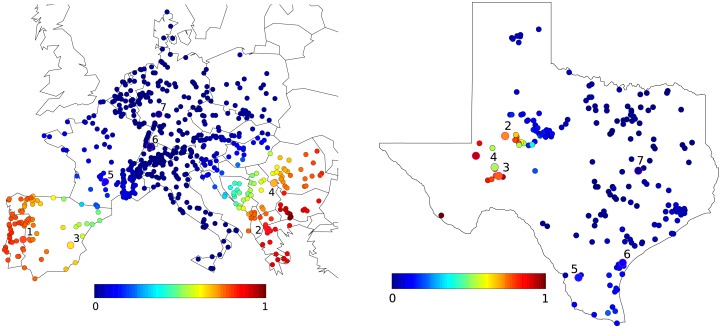
Color plot of the normalized squared components $u_{2k}^{2}$ of the Fiedler mode on generator buses in the European and ERCOT grids. Labeled buses correspond to labeled symbols in Figs 9 and 10.

These results show that, for homogeneous inertia and damping, the short-time RoCoF response *r*_*i*_(*t*) is inversely proportional to the inertia. The behavior at longer times is determined by the magnitude of the few slowest eigenmodes of the network Laplacian on both the perturbation bus [through *u*_*αb*_ in (7)] and the bus where the RoCoF is measured [through *u*_*αi*_]. Despite its neglect of inhomogeneities in inertia and damping, this simple calculation suggests that the so far unexplained behaviors observed in our numerical results in Figs 1, 5 and 6 are related to slow modes of the network Laplacian. This hypothesis gains further support when looking at the structure of the slowest, *α* = 2 mode, so-called Fiedler mode on the generator buses of the European network shown in Fig 8. The large squared amplitude $u_{2i}^{2}$ of the Fiedler mode on buses *i* in Spain and the Balkans is consistent with a disturbance propagating from one to the other of these regions with only minor disturbance on intermediate regions (such as Eastern France in Figs 1, 5 and 6). We have found, but do not show, that the next, *α* = 3 mode has essentially the same profile of $u_{3i}^{2}$ as the Fiedler mode. Furthermore, the next modes *α* = 4, …6 largely avoid Belgium, France, Western Germany, Northern Italy and Switzerland. Higher modes have $\sqrt{\lambda_{\alpha >6}/m-\gamma^{2}/4}\Delta t>4\sqrt{\lambda_{2}/m-\gamma^{2}/4}\Delta t$ (as can be inferred from Fig 7) accordingly, their contribution to RoCoF’s, (7), are at least four times smaller and oscillate four times faster than the Fiedler mode. We therefore neglect them in our qualitative discussions to come.

To assess the disturbance magnitude of a power loss at bus #*b* over frequencies in the whole grid, one needs to gather information on RoCoF’s at different times and locations. We therefore introduce the performance measure

$$\begin{array}{c} M_{b}=\sum_{k=1}^{N{}_{\text{sim}}}\sum_{i}|r_{i}\left(k\Delta t\right)|\;, \end{array}$$ (8)

where *N*_sim_ = 10 is the number of time intervals Δ*t* = 0.5s considered in our numerics. Qualitatively speaking, $M_{b}$ is large, if the RoCoF magnitude |*r*_*i*_| is significant on many nodes and for long times. Fig 2 shows that the total time *N*_sim_Δ*t* = *t*_sim_ = 5 s considered in our numerical calculation of $M_{b}$ is set to include major initial oscillations while neglecting oscillations at longer times of little concern for power grids.

**Fig 9:**
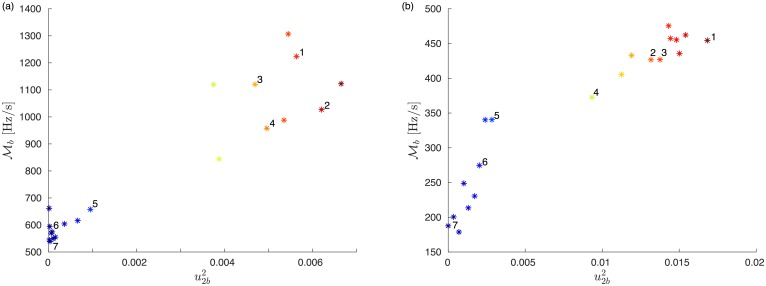
Global RoCoF disturbance magnitude $M_{b}$ as a function of squared Fiedler components $u_{2b}^{2}$ for power losses on 20 different bus #*b* for (a) the European and (b) the ERCOT grid. Labeled symbols correspond to locations indicated in Fig 8. The plots look similar when $M_{b}$ is plotted against the squared component $u_{3b}^{2}$ of the second slowest mode of the Laplacian.

The above result (7) suggests that RoCoF’s are larger following power losses on buses with large components of the eigenvectors with smallest eigenvalues of the Laplacian matrix. To check whether this result also holds in realistic power grids with nonhomogeneous distribution of inertia, we numerically calculate $M_{b}$ for 20 abrupt power losses homogeneously distributed on the European and ERCOT grids. Fig 9 shows that the disturbance magnitude $M_{b}$ grows with the squared Fiedler component $u_{2b}^{2}$ of the location *b* of the power loss. Everything else being kept constant, the disturbance magnitude is more than twice larger in the European grid and almost three times larger in the ERCOT grid for power losses on buses with largest $u_{2b}^{2}$, than for losses on buses with low $u_{2b}^{2}$. As for Fig 8, we have found that the same trend persists when plotting $M_{b}$ against the squared component $u_{3b}^{2}$ of the second slowest mode of the Laplacian. The magnitude of the disturbance following an abrupt power loss is therefore determined by its location, in particular on the amplitude $u_{\alpha b}^{2}$ on the faulted bus *b* of the Fiedler mode (*α* = 2) and of the next slowest mode (*α* = 3) of the network Laplacian. In what follows, we call, by some abuse of language, “Fiedler areas” (“non-Fiedler areas”) the set of buses {*i*} where $u_{2i}^{2}$ and $u_{3i}^{2}$ are large (small).

## 4 Disturbance magnitude vs. inertia

So far we have established that disturbances have strongly location-dependent magnitudes, and in particular that stronger disturbances originate from buses with large amplitude of the two slowest eigenmodes of the network Laplacian. We next investigate how rotational inertia influences this finding. To that end we modify inertia on the network following three different procedures where the inertia of a generator on bus #*i* is increased/decreased according to one of the following probability distributions

$$p_{i}^{U}\propto 1\;,$$ (9)

$$p_{i}^{F}\propto u_{2i}^{2}\;,$$ (10)

$$p_{i}^{\text{nF}}\propto 1/u_{2i}^{2}\;.$$ (11)

The first procedure reduces/adds inertia uniformly (indicated by the superscript ^U^), the second one reduces/adds inertia preferentially on buses with large amplitude of the Fiedler mode (hence the superscript ^F^) and the third one reduces/adds inertia preferentially on buses with small amplitude of the Fiedler mode (with ^nF^ indicating the “non-Fiedler” area).

**Fig 10:**
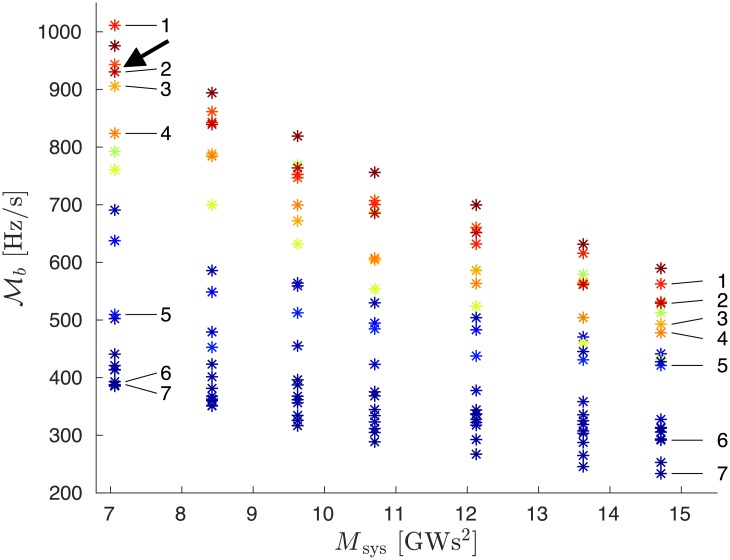
Global RoCoF disturbance magnitude $M_{b}$ vs. the artificially varied system inertia *M*_sys_ in the European grid. Each point corresponds to the loss of a single power station, with colors related to the squared component $u_{2b}^{2}$ of the Fiedler mode on the power loss bus. Color code and label symbols are the same as in Fig 8. The arrow indicates the data point corresponding to the top left data point, also indicated by an arrow in Fig 11.

Fig 10 shows the evolution of $M_{b}$ as a function of total inertia, *M*_sys_ = ∑_*i*_ *m*_*i*_, for power losses of Δ*P* = 900 MW on the same 20 power plants as in Fig 9. The data corresponding to today’s synchronous grid of continental Europe are the rightmost, with the largest amount of inertia. The inertia is then reduced following the first procedure where generator buses become randomly inertialess according to the homogeneous probability distribution (9). One sees that $M_{b}$ follows the ranking defined by the squared Fiedler components, almost regardless of the amount of inertia in the system, and faults in the Fiedler areas are generically more critical than those in the non-Fiedler areas.

**Fig 11:**
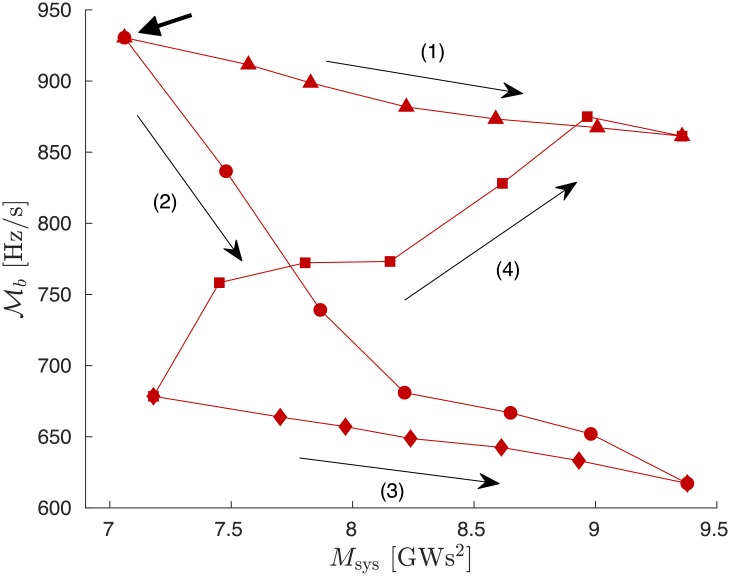
Global RoCoF disturbance magnitude $M_{b}$ vs. artifically modified total system inertia. Along paths (1) and (3), inertia is added according to procedure (11), i.e. mostly on the non-Fiedler area. Path (2) follows procedure (10) by adding inertia almost exclusively on the Fiedler area. Path (4) follows a selected procedure where inertia is removed from the Fiedler area and added on the non-Fiedler area. The top left data point corresponds to the data point indicated by an arrow in Fig 10.

The situation can be dramatically different when following other procedures to add/remove inertia selectively on certain areas. In Fig 11, $M_{b}$ is shown, always for the same power loss. The top left data point (indicated by an arrow) corresponds to the data labeled 2 in the top left of Fig 10 (also indicated by an arrow). Paths (1) and (3) correspond to adding inertia according to procedure (11), i.e. mostly outside the Fiedler area. This procedure reduces $M_{b}$ by less than 10% upon increasing the total inertia *M*_sys_ by 30%. Path (2) follows procedure (10) by adding inertia almost exclusively on the Fiedler area. It is much more efficient and leads to a reduction of $M_{b}$ by more than 30% with the same total increase of *M*_sys_ by 30%. Finally, path (4) illustrates a procedure where inertia is removed from Fiedler areas and added to non-Fiedler areas. In that case, the RoCoF disturbance magnitude increases, even with a global increase of inertia. Taken in reverse direction, path (4) in Fig 11 shows that, quite unexpectedly, grid resilience against faults such as power losses can be enhanced while simultaneously reducing the total amount of inertia.

**Fig 12:**
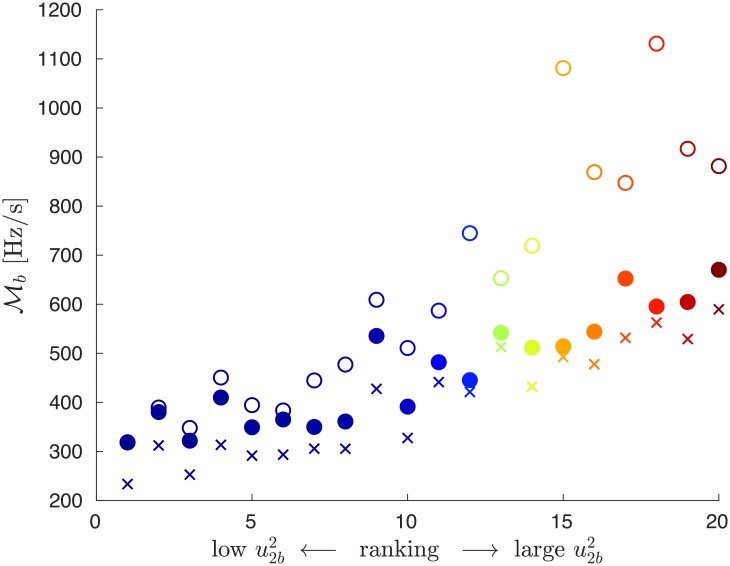
Global RoCoF disturbance magnitude $M_{b}$ horizontally ranked in increasing order of the squared Fiedler mode amplitude $u_{2b}^{2}$ on the faulted bus #*b*. Crosses are for a system with inertia $M_{\text{sys}}^{0}=14.7$ GWs^2^ (corresponding to today’s European grid, Fig 1) and circles for reduced inertia $M_{\text{sys}}=0.6M_{\text{sys}}^{0}$, with system inertia mainly reduced outside Fiedler areas (solid circles) or mainly reduced inside Fiedler areas (empty circles).

We finally show in Fig 12 how global RoCoF disturbance magnitudes depend on the location of each of the 20 power losses considered in Fig 10, once inertia is reduced starting from the full inertia situation of Fig 10 with $M_{\text{sys}}^{0}=14.7$ GWs^2^. The three data sets correspond to unchanged inertia $M_{\text{sys}}^{0}$ (crosses), inertia $M_{\text{sys}}=0.6M_{\text{sys}}^{0}$ reduced mostly in the Fiedler area, following the probability distribution (10) (empty circles) or outside the Fiedler area, according to (11) (full circles). Fig 12 clearly shows that (i) regardless of the position of the fault, inertia reduction on the Fiedler area systematically leads to an enhanced sensitivity to power loss, compared to inertia reduction outside the Fiedler area and (ii) the sensitivity increase is larger for faults on the Fiedler area.

## Conclusion

We have presented numerical investigations on disturbance propagation following a generator fault in the synchronous transmission grid of Continental Europe. The first step was to build up a numerical model, including all necessary parameters to perform dynamical calculations. To the best of our knowledge, no model of this kind is publicly available.

In real power grids, protection devices disconnect generators if the RoCoF or frequency deviations exceed predetermined thresholds. Therefore we based our performance measure on RoCoF and investigated how the latter evolves in space and time following an abrupt power loss, depending on the location of the latter. We have found that disturbances are stronger, they propagate further and persist longer for faults located on areas supporting significant amplitude of the slowest modes of the network Laplacian. In the case of the European grid we found that the two lowest (but nonzero) modes are particularly important in that respect. They have similar geographical support which we called the “Fiedler area”, because the lowest nonzero mode of a network Laplacian is often called the “Fiedler mode”. Amplifying on those results we found that inertia reduction on the Fiedler area leads to an amplified RoCoF response, while reducing the inertia on non-Fiedler area has a much weaker effect, with only a moderate increase of RoCoF’s.

**Fig 13:**
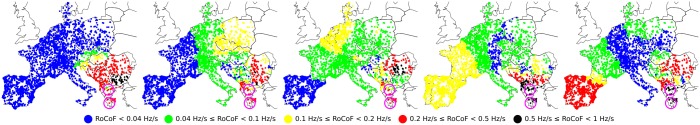
Spatio-temporal evolution of local RoCoFs for two simultaneous abrupt power losses, each of Δ*P* = 1500 MW in a moderate load (and thus low inertia, typical of a standard summer evening) configuration of the synchronous grid of continental Europe of 2018. The faults location is indicated by purple circles. Panels correspond to snapshots over time intervals 0-0.5[s], 0.5-1[s], 1-1.5[s], 1.5-2[s] and 2-2.5[s] from left to right.

**Fig 14:**
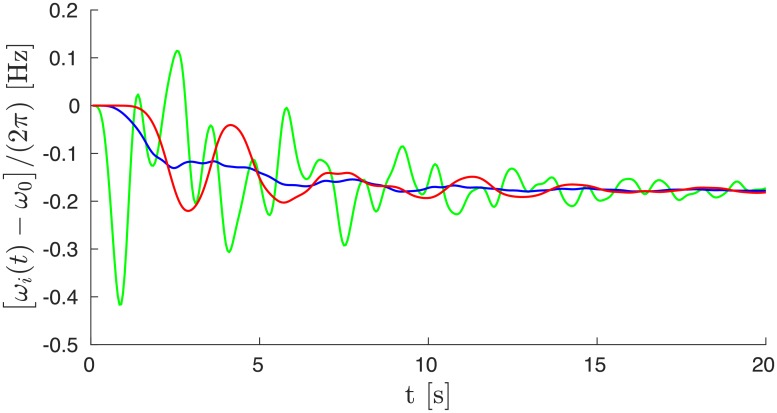
Frequency deviations as a function of time for the double fault of Δ*P* = 3000 MW illustrated in Fig 13, for three buses in the Balkans (green), France (blue) and Spain (red).

The faults considered above correspond to abrupt power losses of Δ*P* = 900 MW. They lead to maximum RoCoF’s magnitudes of 0.5 Hz/s when the fault is located on a Fiedler area under moderate network load conditions. When the fault is located on a non-Fiedler area, RoCoF’s never exceed 0.1 Hz/s. These values are significantly larger when considering a normative contingency of Δ*P* = 3000 MW [14]. This reference incident is usually taken as the tripping of two of the largest European generators, connected to the same bus [14], however since no such generators exist in the Balkans, we show instead in Fig 13 a similar event resulting from the tripping of two nearby 1500 MW power plants, for the same load conditions as in Fig 1. In the ensuing disturbance propagation, RoCoF’s reach values close to 1Hz/s over large areas of south-east Europe for times at least up to 2.5s. Yet, even with a fault of this magnitude, the RoCoF’s are much weaker in France and other non-Fiedler areas than in the Balkans and the spanish peninsula—where the two slowest modes of the network Laplacian reside. Frequency deviations are further shown in Fig 14 which shows the same qualitative, if not quantitative behavior as in Fig 2, but amplified by the more than three times larger fault magnitude, Δ*P* = 900 MW → 3000 MW.

Our findings emphasize an important aspect of optimal inertia location. Because long-range RoCoF disturbance propagation is controlled by the slowest modes of the network Laplacian, reducing inertia where these nodes reside, areas we called “Fiedler areas”, leads to a significantly more sensitive grid than reducing inertia outside these areas. Conversely, substituting inertialess new renewable sources of energy for inertiaful conventional generators critically needs to be accompanied by the deployment of synchronous condensers or synthetic inertia in Fiedler areas, while the need for inertia substitution is lower outside the Fiedler areas. Models similar to our dynamical model for the synchronous grid of continental Europe should prove to be helpful tools in planning for inertia deployment as the penetration of new renewables increases.

As a final comment future works should relate the correlations emphasized in this paper between inertialocation, disturbance propagation and the slow modes of the network Laplacian with those found between the location of inertialess, converter-connected new renewables and specific electromechanical modes in Refs. [4, 5, 23].

## Supporting information

S1 Appendix Disturbance propagation for different faults or with different loads. (PDF)

S2 Appendix A model of the synchronous grid of continental Europe. (PDF)

## References

[1] Machowski J, Bialek J, Bumby JR. Power system dynamics: stability and control. 2nd ed John Wiley & Sons; 2008.

[2] Ulbig A, Borsche TS, Andersson G. Impact of low rotational inertia on power system stability and operation. IFAC Proceedings Volumes. 2014;47(3):7290–7297. 10.3182/20140824-6-ZA-1003.02615

[3] Tielens P, Van Hertem D. The relevance of inertia in power systems. Renewable and Sustainable Energy Reviews. 2016;55:999–1009. 10.1016/j.rser.2015.11.016

[4] Gautam D, Vittal V, Harbour T. Impact of increased penetration of DFIG-based wind turbine generators on transient and small signal stability of power systems. IEEE Transactions on power systems. 2009;24(3):1426–1434. 10.1109/TPWRS.2009.2021234

[5] Eftekharnejad S, Vittal V, Heydt GT, Keel B, Loehr J. Small signal stability assessment of power systems with increased penetration of photovoltaic generation: A case study. IEEE Transactions on Sustainable Energy. 2013;4(4):960–967. 10.1109/TSTE.2013.2259602

[6] Ulbig A, Borsche TS, Andersson G. Analyzing rotational inertia, grid topology and their role for power system stability. IFAC-PapersOnLine. 2015;48(30):541–547. 10.1016/j.ifacol.2015.12.436

[7] Bevrani H, Ise T, Miura Y. Virtual synchronous generators; A survey and new perspectives. Intl Journal of Electrical Power and Energy Systems. 2014;(54):244–254. 10.1016/j.ijepes.2013.07.009

[8] Yan J, Pates R, Mallada E. Performance tradeoffs of dynamically controlled grid-connected inverters in low inertia power systems. In: IEEE 56th Annual Conference on Decision and Control. IEEE; 2017.

[9] Zhong QC, Weiss G. Synchronverters: Inverters that mimic synchronous generators. IEEE Transactions on Industrial Electronics. 2011;58(4):1259–1267. 10.1109/TIE.2010.2048839

[10] Borsche TS, Liu T, Hill DJ. Effects of rotational inertia on power system damping and frequency transients. In: IEEE 54th Annual Conference on Decision and Control. IEEE; 2015. p. 5940–5946.

[11] Poolla BK, Bolognani S, Dörfler F. Optimal placement of virtual inertia in power grids. IEEE Transactions on Automatic Control. 2017;62(12):6209–6220. 10.1109/TAC.2017.2703302

[12] Pirani M, Simpson-Porco JW, Fidan B. System-theoretic performance metrics for low-inertia stability of power networks. In: Decision and Control (CDC), 2017 IEEE 56th Annual Conference on. IEEE; 2017. p. 5106–5111.

[13] Borsche TS, Dörfler. On placement of synthetic inertia with explicit time-domain constraints. arXiv:170503244. 2017.

[14] ENTSO-E. Frequency Stability Evaluation Criteria for the Synchronous Zone of Continental Europe; 2016. https://docs.entsoe.eu/dataset/inertia-report-continental-europe/.

[15] Siami M, Motee N. Fundamental limits and tradeoffs on disturbance propagation in linear dynamical networks. IEEE Transactions on Automatic Control. 2016;61(12):4055–4062. 10.1109/TAC.2016.2547982

[16] Wolter J, Lünsmann B, Zhang X, Schröder M, Timme M. Quantifying transient spreading dynamics on networks. arXiv:171009687. 2017. 10.1063/1.5000996 29960404

[17] Tamrakar S, Conrath M, Kettemann S. Propagation of Disturbances in AC Electricity Grids. Scientific Reports. 2018;8:6459 10.1038/s41598-018-24685-5 29691445 PMC5915393

[18] Birchfield AB, Xu T, Gegner KM, Shetye KS, Overbye TJ. Grid structural characteristics as validation criteria for synthetic networks. IEEE Transactions on power systems. 2017;32(4):3258–3265. 10.1109/TPWRS.2016.2616385

[19] Bergen AR, Hill DJ. A structure preserving model for power system stability analysis. IEEE Transactions on Power Apparatus and Systems. 1981;(1):25–35. 10.1109/TPAS.1981.316883

[20] Tyloo M, Coletta T, Jacquod P. Robustness of synchrony in complex networks and generalized Kirchhoff indices. Physical Review Letters. 2018;(120):084101 10.1103/PhysRevLett.120.084101 29542999

[21] Tyloo M, Pagnier L, Jacquod P. The key player problem in complex oscillator networks and electric power grids: resistance centralities identify local vulnerabilities. to be published. 2018. 10.1126/sciadv.aaw8359 PMC6874484 31803830

[22] Jenkins N, Allan R, Crossley P, Kirschen D, Strbac G. Embedded generation. The Institution of Engineering and Technology; 2000.

[23] Quintero J, Vittal V, Heydt GT, Zhang H. The impact of increased penetration of converter control-based generators on power system modes of oscillation. IEEE Transactions on Power Systems. 2014;29(5):2248–2256. 10.1109/TPWRS.2014.2303293

