# Supplementary material for: Inertia location and slow network modes determine disturbance propagation in large-scale power grids
Source: PLoS One. 2019 Mar 21;14(3):e0213550. doi: 10.1371/journal.pone.0213550 (PMC6428310; doi:10.1371/journal.pone.0213550)
Supplement: S1 Appendix — (PDF) [file pone.0213550.s001.pdf]

Supporting information to: Inertia location and slow network modes determine disturbance propagation in large-scale power grids

Laurent Pagnier, Philippe Jacquod

S1 Appendix. Disturbance Propagation for different faults or with different loads.

We present additional data complementing those in Figs. 1, 5 and 6 on disturbance propagation following a power loss.

Fig. A shows disturbance propagation following different power losses for the same load and the same European grid as in Fig. 1. Fig. B shows disturbance propagation following the same power losses as in Fig. 1, but this time on a more loaded grid corresponding to a winter evening. Because of the larger load, more generators are working and therefore the total effective inertia on the grid is larger. Disturbance propagation in both instances exhibit the same qualitative behavior as in Fig. 1, with quantitative differences arising at short times for Fig. B with more inertia (as expected).

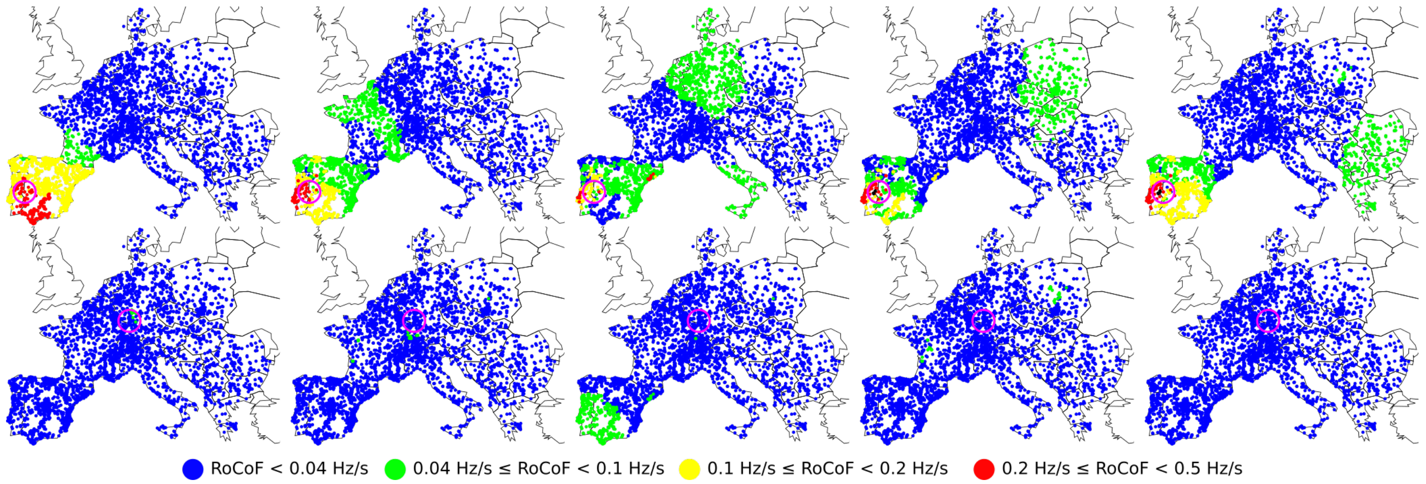

Fig A. Spatio-temporal evolution of local RoCoFs for two different power losses of $\Delta P = 900$ MW. in a moderate load (typical of a standard summer evening) configuration of the synchronous grid of continental Europe of 2018. The top four panels correspond to a fault in Spain and the bottom four to a fault in Western Germany. In both cases, the fault location is indicated by a purple circle. Panels correspond to snapshots over time intervals 0-0.5[s], 0.5-1[s], 1-1.5[s], 1.5-2[s] and 2-2.5[s] from left to right.

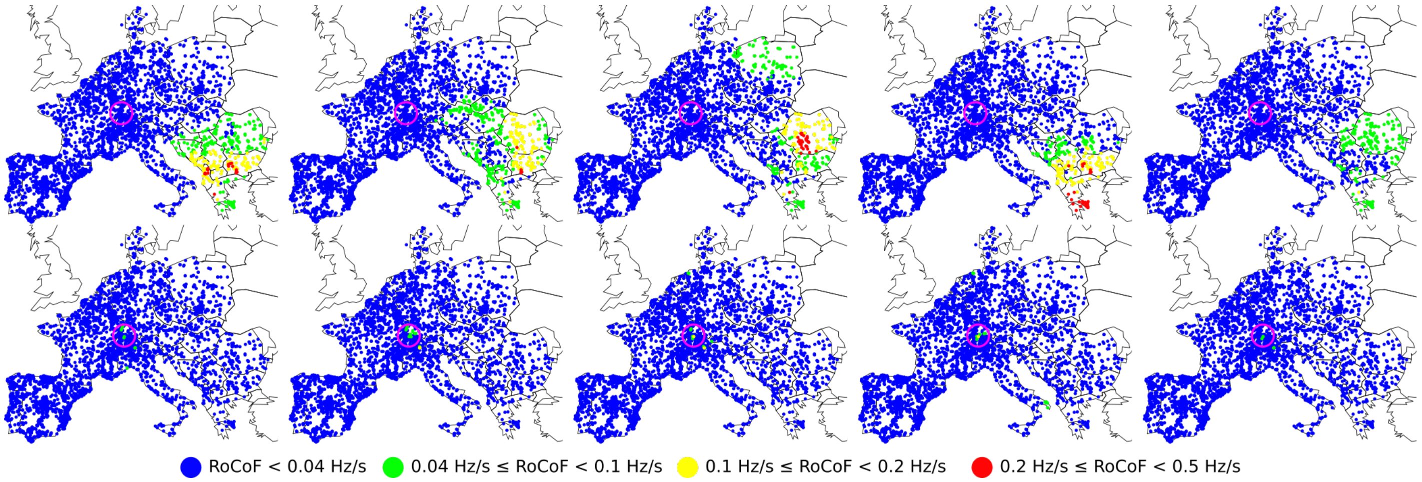

Fig B. Spatio-temporal evolution of local RoCoFs for the same two abrupt power losses of $\Delta P = 900$ MW as in Fig. 1 but with a higher load (typical of an early evening in winter) configuration. The top four panels correspond to a fault in Greece and the bottom four to a fault in Switzerland. In both cases, the fault location is indicated by a purple circle. Panels correspond to snapshots over time intervals 0-0.5[s], 0.5-1[s], 1-1.5[s], 1.5-2[s] and 2-2.5[s] from left to right.
