# Supplementary material for: Inertia location and slow network modes determine disturbance propagation in large-scale power grids
Source: PLoS One. 2019 Mar 21;14(3):e0213550. doi: 10.1371/journal.pone.0213550 (PMC6428310; doi:10.1371/journal.pone.0213550)
Supplement: S2 Appendix — (PDF) [file pone.0213550.s002.pdf]

Supporting information to: Inertia location and slow network modes determine disturbance propagation in large-scale power grids

Laurent Pagnier, Philippe Jacquod

S2 Appendix. A model of the synchronous grid of continental Europe.

A. Publicly available models

There are only few publicly available models of part or all of the synchronous grid of continental Europe. To the best of our knowledge, the first one was released by Zhou and Bialek [S1] and later upgraded to incorporate the Balkans [S2]. Other models include ELMOD [S3], PEGASE [S4, S5] and PyPSA-Eur [S6]. These useful power flow / optimal power flow models have not been extended to dynamical simulations and, except PyPSA-Eur, they lack bus geolocalization. We have constructed our own model to circumvent these two shortcomings. We briefly describe how this construction proceeded.

B. Geolocalized model for dynamical simulations of the continental European grid

We built our grid model from different publicly available databases under Creative Commons licenses. Similar building procedures were used in Refs. [S1, S3]. A similar model has recently been constructed, whose parameters do not seem to be publicly available [S7].

1) Geolocalization of buses and lines: Wiegmans has extracted geolocalization data for the continental European grid from the ENTSO-E interactive map [S8]. Our starting point is his database, which contains location and voltage of buses, identified as either generator or load buses, length and voltages for transmission lines and voltages for transformers. We determined the principal component of that grid and discarded non-connected buses. The final network has 3809 buses connected by 4944 transmission lines.

2) Electrical parameters of transmission lines: Transmission lines operate at two different voltages of either 220 kV or 380 kV. Within the lossless line approximation used in this manuscript, lines have purely imaginary admittances and are therefore characterized by their susceptance B_{ij} [S9]. They are given by

$$B_{ij} = 1/(X_{ij} l_{ij}) \,, \tag{S1}$$

where l_{ij} is the length of the line (measured in kilometers) and X_{ij} is its kilometric reactance. For the latter we use $X_{ij} = 360\,\mathrm{m}\Omega/\mathrm{km}$ for 220 kV lines and $X_{ij} = 265\,\mathrm{m}\Omega/\mathrm{km}$ for 380 kV lines. These values correspond to averages of those found in Ref. [S10].

3) Distribution of national loads: Time series for national loads are available for member countries of ENTSO-E [S11]. For each country, we distribute those loads demographically over the set of consumer buses [S1, S3].

Geographical population distributions are first determined from the GeoNames database [S12]. Second, the population of each town is distributed over all buses that are less than $d_{\max} = 50$ km away from it proportionally to their weight $w = 1$ for 220 kV buses and $w = 3$ for 380 kV buses. This determines the effective population attributed to each bus. Third, the national load is distributed to each national bus in

proportion to their attributed population. The validity of the procedure is at least partly confirmed by the strong correlation between population and load distributions in Italy reported in Ref. [S1].

4) Conventional generators: capacity and dispatch: Wiegmans' database contains partial information on generator types and rated power [S8]. The missing generator data are obtained from the global energy observatory website [S13]. Fig. C compares the national installed capacities for some European countries in our model to those listed in Ref. [S11].

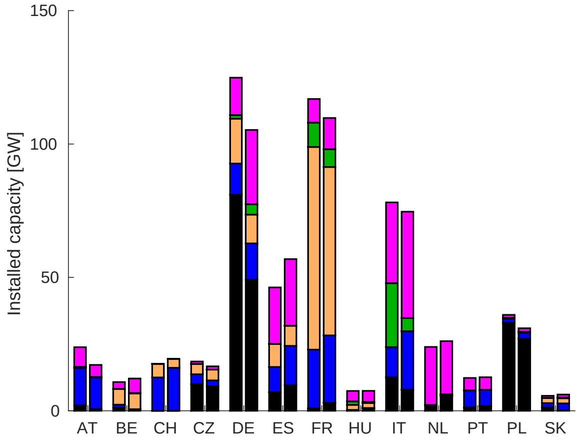

Fig C. Installed capacity according to our grid model (left column) and to Ref. [S11] (right column) for several European countries.

Given distributed loads, we use an economic dispatch in the form of a DC optimal power flow to obtain power feed-in from conventional generators. Table A gives the marginal costs c_i that we use for different technologies.

Table A. Marginal cost and inertia constant for different types of conventional generators.

	c_i [\$/MWh]	H_i [s]
Hydro	80	4
Nuclear	16	6
Lignite	16	6
Hard coal	35	6
Gas	100	6
Other	7	3

5) Conventional generators: inertia and damping coefficients: Rotating machines are characterized by their inertia constant H_i which is the time over which the rated power of the generator provides a work equivalent to its kinetic energy [S9, S14]. It is related to the inertia coefficient in (2) via

$$m_i = \frac{2H_i P_i^{(0)}}{\omega_0}\,, \tag{S2}$$

where $P_i^{(0)}$ is the rated power of the generator. Table A summarizes the values of inertia constants we use in our grid model [S15]. Together with (S2) and the rated powers obtained in the previous paragraph, they determine the inertia coefficients m_i for each generator type.

Damping coefficients d_i are finally obtained for each generator type from Eq. (5.24) and Table 4.3 in Ref. [S14].

6) Frequency dependent loads: In the structure-preserving model we are using, loads are frequency-dependent [S16]. The load frequency/damping coefficient d_i can be expressed as

$$d_i = \frac{\alpha P_i^0}{\omega_0}\,. \tag{S3}$$

with the load P_i^0 at nominal frequency ω_0. The frequency-dependence of loads has been experimentally investigated [S17, S18], with reported values $\alpha \in 0.8 - 2$. In this manuscript, we use $\alpha = 1.5$.

References

[S1] Zhou Q, Bialek JW. Approximate model of European interconnected system as a benchmark system to study effects of cross-border trades. IEEE Transactions on power systems. 2005;20(2):782–788.

[S2] Hutcheon N, Bialek JW. Updated and validated power flow model of the main continental european transmission network. In: PowerTech, 2013 IEEE Grenoble. IEEE; 2013.

[S3] Leuthold FU, Weigt H, Von Hirschhausen C. A large-scale spatial optimization model of the european electricity market. Networks and spatial economics. 2012;12(1):75–107.

[S4] Josz C, Fliscounakis S, Maeght J, Panciatici P. AC power flow data in MATPOWER and QCQP format: iTesla, RTE snapshots, and PEGASE. arXiv:160301533. 2016;.

[S5] Zimmerman R, Murillo-Sánchez C, Thomas RT. MATPOWER: Steady-State Operations, Planning and Analysis Tools for Power Systems Research and Education. IEEE Trans on Power Systems. 2011;26:12–19.

[S6] Hörsch J, Hofmann F, Schlachberger D, Brown T. PyPSA-Eur: An open optimisation model of the European transmission system. Energy Strategy Reviews. 2018;22:207–215.

[S7] Hewes D, Altschaeffl S, Boiarchuk I, Witzmann R. Development of a dynamic model of the European transmission system using publicly available data. In: Energy Conference (ENERGYCON), 2016 IEEE International. IEEE; 2016. p. 1–6.

[S8] Wiegmans B. GridKit extract of ENTSO-E interactive map; 2016. https://doi.org/10.5281/zenodo.55853.

[S9] Bergen AR, Vittal V. Power Systems Analysis. 2nd ed. Pearson/Prentice Hall; 2000.

[S10] Power Engineering Guide; 2014. http://siemens.com/energy/peg.

[S11] ENTSO-E. ENTSO-E Transparency platform; 2015. https://transparency.entsoe.eu/.

[S12] Geonames. Cites1000 data base;. http://download.geonames.org/export/.

[S13] Global energy observatory. GEO Power plants database;. http://globalenergyobservatory.org/.

[S14] Machowski J, Bialek J, Bumby JR. Power system dynamics: stability and control. 2nd ed. John Wiley & Sons; 2008.

[S15] Kundur P, Balu NJ, Lauby MG. Power system stability and control. McGraw-hill New York; 1994.

[S16] Bergen AR, Hill DJ. A structure preserving model for power system stability analysis. IEEE Transactions on Power Apparatus and Systems. 1981;(1):25–35.

[S17] Welfonder E, Weber H, Hall B. Investigations of the frequency and voltage dependence of load part systems using a digital self-acting measuring and identification system. IEEE Transactions on Power Systems. 1989;4(1):19–25.

[S18] O'Sullivan J, O'Malley M. Identification and validation of dynamic global load model parameters for use in power system frequency simulations. IEEE Transactions on Power Systems. 1996;11(2):851–857.
